# Oxaliplatin-induced Evan's syndrome

**DOI:** 10.1054/bjoc.2000.1608

**Published:** 2001-02

**Authors:** C C Earle, W Y Chen, D P Ryan, R J Mayer

**Affiliations:** Department of Adult Oncology, Dana-Farber Cancer Institute, Department of Medicine, Brigham and Women's Hospital, Boston, MA, 02115, USA; Division of Hematology/Oncology, Department of Medicine, Massachusetts General Hospital, 32 Fruit St., Boston, MA, 02114, USA; Harvard Medical School, Boston, MA, 02115, USA


*British Journal of Cancer* (2001) **84**(3), 441
© 2001 Cancer Research Campaign
doi: 10.1054/ bjoc.2000.1608, available online at http://www.idealibrary.com on IDEAL®

Letter to the Editor

### Oxaliplatin-induced Evan's syndrome

**Sir,**

Oxaliplatin is a third-generation platinum compound with activity in advanced colorectal cancer. It is available in several European countries, but remains investigational in North America. Its safety profile compares favourably with the other platinum-based agents, cisplatin and carboplatin, and consists mostly of peripheral neuropathy and mild thrombocytopenia. However, there has been one previous report in the literature of fatal haemolytic anaemia caused by oxaliplatin (Desrame et al, 1999). Here we report immune-mediated thrombocytopenia and concurrent haemolytic anaemia (Evan's syndrome) that developed during oxaliplatin infusion.

In November of 1999, a 56-year-old man with a 2-year history of colon cancer metastatic to liver and lung, who had progressed on first-line 5-FU and leucovorin as well as second-line irinotecan, was enrolled on a Phase II study at the Dana-Farber Cancer Institute. Treatment consisted of oxaliplatin 85 mg/m$^2$ by bolus infusion over 2 hours, followed by 500 mg/m$^2$ of leucovorin also infused over 2 hours, 5-FU 400 mg/m$^2$ by rapid venous infusion, and then 5-FU 2400 mg/m$^2$ by continuous infusion with a portable pump over 46 hours. Treatment was repeated every 2 weeks. The patient had stable disease on this regimen between November 1999 and July 2000.

In July, the patient came for a routine treatment with a platelet count of 99 000 $\mu l^{-1}$ immediately prior to infusion. During the leucovorin infusion, approximately 5 hours after the baseline bloodwork was drawn and the oxaliplatin infusion initiated, he began feeling unwell and his gums began to bleed. He subsequently developed purpura on his oral mucosa and eyelids, haematuria and haematochezia. A repeat platelet count was 6000 $\mu l^{-1}$.He remained haemodynamically stable throughout, and initial haemoglobin and INR were normal. He was admitted to hospital for platelet transfusion and supportive care, but quickly also developed anaemia with a drop in his haemoglobin from 108 to 78 g $l^{-1.}$ Peripheral smear confirmed the decreased platelets, with a left shift in red cell morphology. There was anisocytosis and polychromasia, with a high RDW of 17.3 and increased reticulocytes at 4.7%. Bilirubin rose to 4.4 mg $dl^{-1}$, with only 1.4 mg $dl^{-1}$ being direct, and the LDH increased to 2148 U $l^{-1.}$ Laboratory testing indicated a warm antibody, with a positive direct Coomb's test with polyspecific IgG. There was no anti-C3 antibody, and the eluate reacted with all cells. His INR also rose transiently to 2.4. Clotting factor levels and fibrinogen were normal, although Fibrin Split Products were elevated at 8 $\mu g\ ml^{-1}$. Screening for heparin-induced thrombocytopenia and thrombosis (HITT) was negative.

The patient was treated with prednisone 75 mg orally per day, with stabilization of his blood indices over the course of 3 days after receiving a total of 4 units of single donor platelets, 3 units of packed red blood cells, and 4 units of fresh frozen plasma. He made a complete clinical recovery, with a platelet count of 122 000 $\mu l^{-1}$ and a hemoglobin of 109 g $l^{-1}$ 2 weeks later while tapering off the steroids. In the month since this episode he has had continued disease stability, but he has not been re-challenged with oxaliplatin.

Haemolytic anaemia has previously been reported with cisplatin (Levi et al, 1981) and carboplatin (Marani et al, 1996), as well as oxaliplatin. In contrast to the previous case report of oxaliplatin-mediated haemolytic anaemia, our patient recovered with supportive treatment. The lack of anti-C3 and the fact that the eluate reacted with all cells is less consistent with a drug-induced haemolysis, but the timing and rapidity with which the thrombocytopenia developed is highly suggestive of an immune-mediated cause. To our knowledge there have been no previous reports of immune-mediated platelet destruction due to oxaliplatin associated with, and preceding, haemolytic anaemia.

*CC Earle[1,3], WY Chen[1,3], DP Ryan[2,3] and RJ Mayer[1,3]*
*[1]Department of Adult Oncology, Dana-Farber Cancer Institute, Department of Medicine, Brigham and Women's Hospital, Boston, MA 02115, USA;*
*[2]Division of Hematology/Oncology, Department of Medicine, Massachusetts General Hospital, 32 Fruit St., Boston, MA 02114, USA;*
*[3] Harvard Medical School, Boston, MA 02115*

### REFERENCES

Desrame J et al (1999) Oxaliplatin-induced hemolytic anemia. *Lancet* **354**: 1179–1180

Levi JA, et al (1981) Haemolytic anaemia after cisplatin treatment. *BMJ* **282**: 2003–2004

Marani TM, et al (1996) Carboplatin-induced immune hemolytic anemia. *Transfusion* **36**: 1016–1018

